# Supplementary material for: Exposed soil and mineral map of the Australian continent revealing the land at its barest
Source: Nat Commun. 2019 Nov 22;10:5297. doi: 10.1038/s41467-019-13276-1 (PMC6874580; doi:10.1038/s41467-019-13276-1)
Supplement: Supplementary file 1 — Supplementary Information [file 41467_2019_13276_MOESM1_ESM.pdf]

Supplementary Information

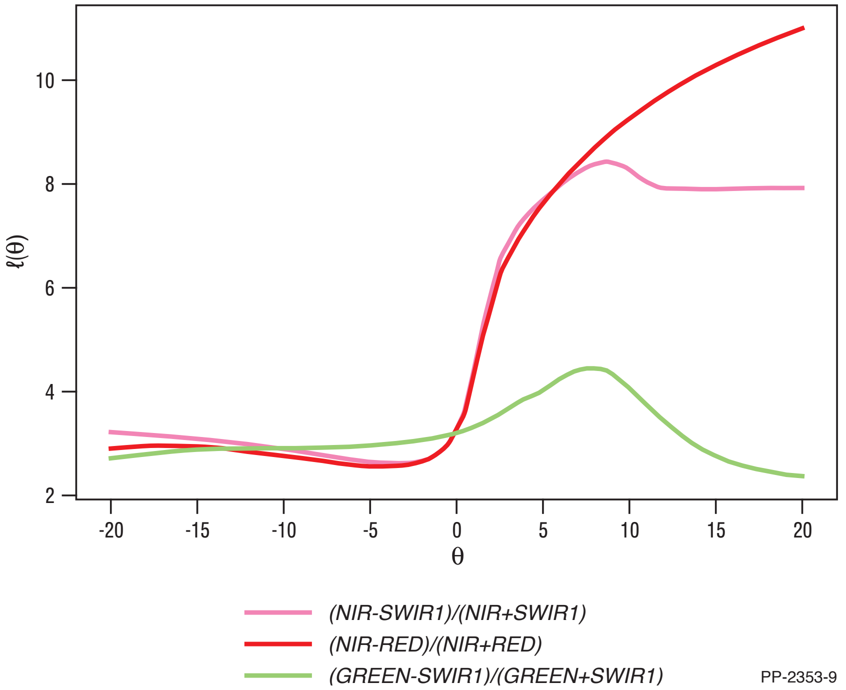

Supplementary Figure 1. Impact of the weight parameter on various loss functions. The loss function ℓ plotted for 3 simple choices of one feature models. We observe a smooth relationship between the value of the loss function $\ell(\theta)$ (y-axis) and the model weight parameter $\theta = (c_1)$ (x-axis) allowing the minimal loss to be easily identified. For example, for the $\tilde{w}(\theta) = c_1\ (\mathrm{NIR} - \mathrm{RED})/(\mathrm{NIR} + \mathrm{RED})$ weight model (red line) the minimum value for $\theta = (c_1)$ is at $c_1 \approx -3$.

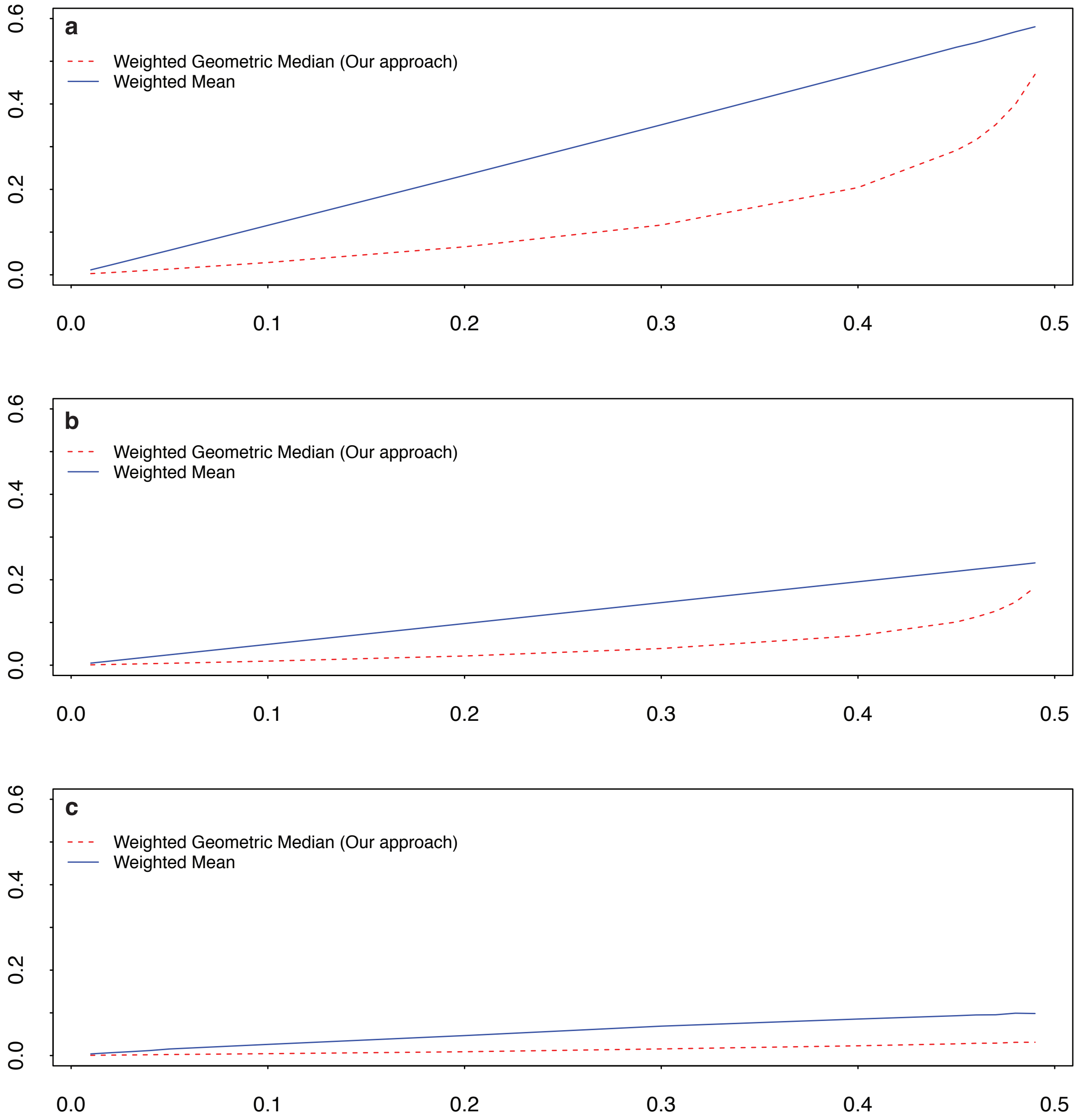

Supplementary Figure 2. Simulation study showing the robustness of our algorithm to noise and corruption. (**a**) Simulation study where generated $n = 1000$ synthetic surface reflectance observations (as a value between 0 and 1 in each wavelength) of dimension $p = 6$ with a mean of $\mu = (0.5, 0.5, 0.5, 0.5, 0.5, 0.5)$ and covariance matrix with an AR(1) structure given by $\Sigma = \sigma \times (\rho^{|i-j|})$ for $1 \le i, j \le p$, where we chose $\rho = 0.3$ and $\sigma = 0.01$. In the x-axis, we show the percentage corruption where the corruption is given by setting observations to (1,1,1,1,1,1). The y-axis gives the Euclidean distance between the initial (uncorrupted) output and the corrupted output (i.e., the deviation) shown in a dashed red line. We plot up to the breakdown point of the geometric median which is 0.5. We compare to the same model but with the weighted geometric median replaced by a weighted mean and plot its deviation as well (shown in a blue solid line). (**b**) We show similar metrics where the noisy perturbations are given by parallel shifts of $x + (0.2, 0.2, 0.2, 0.2, 0.2, 0.2)$ to the surface reflectance x. (**c**) The case where perturbations are given by changing the spectra to a random vector sampled from the truncated Normal distribution with mean μ and covariance $\Sigma = \mathrm{diag}(0.04, \ldots, 0.04)$ where the truncation bounds are 0 and 1 to ensure that the vectors are given in terms of surface reflectance. All three plots show a significant robustness of our approach compared to a similar approach where the weighted mean is used. The deviation curve, as a function of the percentage of corruption, shows strong resilience.
